# Editorial: Physiological growth of crops in saline-alkali land and its new quality productive control methods

**DOI:** 10.3389/fpls.2026.1784181

**Published:** 2026-02-23

**Authors:** Praveen Guleria, Junliang Fan, Lijun Su, Jihong Zhang, Wenling Chen

**Affiliations:** 1 Plant Biotechnology and Genetic Engineering Lab, Department of Biotechnology, DAV University, Jalandhar, India; 2 Northwest A&F University, Xianyang, China; 3 Xi’an University of Technology, Xi’an, China; 4 Shihezi University, Shihezi, China; 5 China University of Geosciences Wuhan, Wuhan, China

**Keywords:** irrigation, metabolomics, nutrient bioavailability, soil-water relation, spatial distribution, transcriptomics

## Introduction

The rising human population has induced undue pressure on agricultural sustainability in the face of changing climatic conditions, resource limitations, and altered soil parameters. Among various abiotic stresses affecting sustainable crop productivity, salinity and alkalinity of the soil are the most rapidly expanding risks to global food security. Both stresses negatively affect the soil structure, reduce the bioavailability of nutrients, and induce several physiological changes to the plants, thus causing significant yield losses across various agroecosystems. As the global soil and agricultural land are expected to be severely affected by salinity and alkalinity in the coming years, there is an urgency to devise sustainable agricultural strategies for improved crop productivity to meet the food requirements of a continuously rising population. Given this, this Research Topic was launched for the understanding of strategies and mechanisms adopted by crops for the mitigation of enhanced salinity and alkalinity in the soil.

## Strategies for salinity-alkalinity mitigation

Saline and alkaline soils induce several devastating effects on the growth, development, and productivity of plants via interconnected complex mechanisms. Soil salinity poses enhanced salt toxicity to plants, thus causing ionic and osmotic imbalance. Alkaline soil conditions, on the other hand, inhibit its aggregation potential, reduce the bioavailability of macronutrients and micronutrients, and hinder plant-microbial interaction efficiency. Ma et al. investigated the spatiotemporal distribution of soil salinity and nutrient bioavailability in the mild saline soil of the Yellow River in the Shandong Province of China in October 2020 and April 2021. Salinity was observed to be higher during April than during October due to enhanced variability of nitrogen, potassium, and phosphorus in the soil sampled. Salinity and potassium during October showed weak spatial dependence, whereas nitrogen and potassium in April showed moderate spatial dependence. Therefore, seasonal variation of nutrient availability induced soil-mediated stress, which can be balanced with exogenous fertilizer supply.

An important prerequisite to this is the understanding of soil spatial tendency, soil-water relations, and water-nutrient management. Various statistical tools, like kriging, have helped in understanding the spatial and temporal variation of water, salinity, alkalinity, and nutrient distribution in soil across various climatic zones (Li and Heap, 2011). Fu et al. and Zhang et al. evidenced the potential of a theoretical simulation tool like AquaCrop for the development of an appropriate irrigation system to sustain soil water availability with accuracy. AquaCrop accurately simulated the crop canopy, their biomass, soil-water relation, and ultimately the crop yield of wheat and maize across seasonal variation from 2022 to 2024. Further, nutrient availability was identified to be dependent on soil water relations in agricultural lands. Wang et al. investigated the mechanisms underlying irrigation and nitrogen supply-mediated regulation of nitrogen accumulation and root growth in cotton. The cotton root diameter was significantly related to the water irrigation schedule rather than the nitrogen fertilizer input. The nitrogen fertilizer was identified as a nitrogen source to the soil, and later, the irrigation quota was responsible for the efficient nitrogen accumulation in plants. Another significant strategy for efficient water and nutrient utilization is mixed cropping. Li et al. optimized the inter-cropping of wolfberry with alfalfa in arid saline-alkaline soil as an efficient water control strategy to enhance their growth, photosynthesis, and productivity with sustainable utilization of water resources.

## Mechanism of saline-alkaline stress regulation

With the advent of advanced techniques and technologies, the physiological and molecular mechanisms underlying the plant responses to salty and alkaline soils are being elucidated. Pan et al. investigated and revealed the molecular mechanism of salt-alkali stress mitigation of *Cerasus humilis*. The genes *ChWRKY29* and *ChWRKY34* were observed to enhance the transcript accumulation of *ChMSD2* and *ChCSD2* on alkali exposure, thus increasing the antioxidant potential of plants. Further, genome-wide bioinformatic analysis revealed 9 *ChSOD* genes in *C. humili*, with their promoter elements responsible for inducing abiotic stress resistance in plants. Wang et al. revealed 7355 differentially expressed genes in soybean responding to salt and alkaline stress using transcriptome analysis. The genes upregulated to these stresses were identified to be involved in phenylpropanoid biosynthesis and hormone signaling. In addition, transcription factors *MYB*, *ERF*, *bHLH*, and *WRKY* showed internetworking during stress mitigation. Efficient Na^+^ sequestration in the roots and regulated expression of genes were responsible for salt-alkali stress mitigation in soybean. Geng et al. have also revealed 6610 differentially expressed genes and 579 transcription factors involved in the regulation of phenylalanine metabolism, linolenic acid metabolism, and flavonoid biosynthesis for salt-alkali stress mitigation of cotton. Moreover, 4225 metabolites and 1684 differentially accumulated metabolites were also identified. The combined transcriptomic and metabolomic analysis identified flavonoid metabolism and linolenic acid metabolism as the key regulatory pathways for alkali tolerance. Further, depending on abiotic stress-resistant rootstock is also a considerable strategy to optimize the nitrogen utilization of plants. Song et al. explored the molecular mechanism behind enhanced nitrogen utilization efficiency of peach on grafting with stress resistant GF677 rootstock. The upregulation of nitrogen metabolism and antioxidant responsive genes, and precisely *PpNRT1*, *PpNRT4*, *PpGLDH*, and *PpGRF9* were validated to enhance nitrogen utilization; and *PpMGrx2* was responsible for the alkaline stress mitigation.

## Concluding remarks

Plant stress management is not only physiological but is also significantly regulated by molecular mechanisms and inter-networks. Identification of the physiological and molecular aspects of plant stress management is a potential target for future crop research in the development of saline-alkaline resistant plants using plant breeding and biotechnology. It is therefore important to understand that plants are being exposed to the combined effects of salinity and alkalinity under natural field conditions. So, to ensure sustainable plant mitigation responses, current research should be focused on elucidating distinctive as well as interactive plant responses to the individual and concomitant saline and alkaline stress. Addressing this can be a fundamental step towards more targeted and sustainable management of saline-alkaline soils. As soil salinity and alkalinity continue to expand around the globe, the incremental improvements can no longer be sufficient and sustainable. Hence, holistic and research-driven strategies are the requisites to ensure agricultural productivity and food security in the era of accelerating global climate change.
